# Raw Onions as an Hypnotic

**Published:** 1876-09

**Authors:** 


RAW ONIONS AS AN HYPNOTIC.—A French writer professes to have made a valuable discovery in regard to the soporific power of raw onions. After complaining of the unpleasant after-effects of opiates, he says of onions:

"I administer them to myself, and I have often prescribed them for others; and their action is manifest, whether taken raw or in sauce. Every one knows the taste of the onion, which it derives from a peculiar essential oil contained in the coats of this precious and wholesome bulb. This essence possesses, I am sure, powerful soporific virtues, which, in my hands, never fail to be realized. When I am weary with labor and feel little inclined to sleep, I eat two or three little onions, and the effect is magical. This is also an excellent ailment for persons long exposed to intense cold."

The editor of *Le Mouvement Medical*, commenting on the statement, thinks the odor of an onion-eater's breath would not act as an anodyne on him, in which respect he does not stand alone. In the early settlement of California, onions were in great demand among the miners, who would pay two or three dollars for one. But they were used as an anti-scorbutic, and when sliced, and seasoned with salt, pepper, and vinegar, were deemed a luxury. Potatoes also were purchased at enormous prices, and eaten raw, like apples, for the same purpose. —*Pacific Med. and Surg. Jour.*

